# Low and Borderline Ankle–Brachial Index Is Associated With Intracranial Aneurysms: A Retrospective Cohort Study

**DOI:** 10.1227/neu.0000000000002837

**Published:** 2024-01-25

**Authors:** Dan Laukka, Essi Kangas, Aino Kuusela, Jussi Hirvonen, Tiia Rissanen, Melissa Rahi, Juri Kivelev, Ville Rantasalo, Maarit Venermo, Jaakko Rinne, Harri Hakovirta

**Affiliations:** * Department of Neurosurgery, Neurocenter, Turku University Hospital, Turku, Finland;; ‡ Clinical Neurosciences, University of Turku, Turku, Finland;; § Department of Surgery, University of Turku, Turku, Finland;; ‖ Department of Vascular Surgery, Turku University Hospital, Turku, Finland;; ¶ Department of Radiology, Turku University Hospital and University of Turku, Turku, Finland;; # Department of Radiology, University of Tampere, Tampere, Finland;; ** Department of Clinical Medicine, Biostatistics, University of Turku, Turku, Finland;; ‡‡ Department of Biostatistics, University of Turku and Turku University Hospital, Turku, Finland;; §§ Department of Vascular Surgery, University of Helsinki and Helsinki University Hospital, Helsinki, Finland;; ‖‖ Department of Surgery, Satasairaala, Pori, Finland

**Keywords:** Ankle–brachial index, Intracranial aneurysm, Peripheral arterial disease, Prevalence, Risk factor

## Abstract

**BACKGROUND AND OBJECTIVES:**

A low ankle–brachial index (ABI) has been linked to systemic inflammation and an elevated risk of cardiovascular events, most notably myocardial infarction and stroke. Intracranial aneurysms (IAs) share similar risk factors with other cardiovascular diseases. However, the association between low ABI and IAs has not been sufficiently investigated. Our objective was to investigate the potential connection between ABI values and the prevalence of unruptured IAs.

**METHODS:**

This retrospective cohort study reviewed 2751 patients who had ABI measurements at a public tertiary hospital from January 2011 to December 2013. Patients with available cerebrovascular imaging or a diagnosis of ruptured IA were included in the study (n = 776) to examine the association between ABI and saccular IAs. The patients were classified into 4 groups: low ABI (≤0.9, n = 464), borderline ABI (0.91-0.99; n = 47), high ABI (>1.4, n = 57), and normal ABI (1.00-1.40; n = 208).

**RESULTS:**

The prevalence of IAs was 20.3% (18.1% unruptured IAs) in the low ABI group, 14.9% (12.8% unruptured IAs) in the borderline ABI group, 7.0% (5.3% unruptured IAs) in the high ABI group, and 2.4% (1.9% unruptured IAs) in the normal ABI group (*P <* .001). There were no significant differences in the prevalence of ruptured IAs between the ABI groups (*P* = .277). Sex- and age-adjusted multinomial regression, including clinically relevant variables, revealed that low ABI (odds ratio [OR], 13.02; 95% CI, 4.01-42.24), borderline ABI (OR, 8.68; 95% CI, 2.05-36.69), and smoking history (OR, 2.01; 95% CI, 1.07-3.77) were associated with unruptured IAs.

**CONCLUSION:**

The prevalence of unruptured IAs was 9-fold higher in the low ABI group and nearly 7-fold higher in the borderline ABI group when compared with the normal ABI group. ABI measurements could be clinically relevant for identifying individuals at higher risk of IAs and may help guide screening and preventive strategies.

## Graphical abstract

**Figure:**
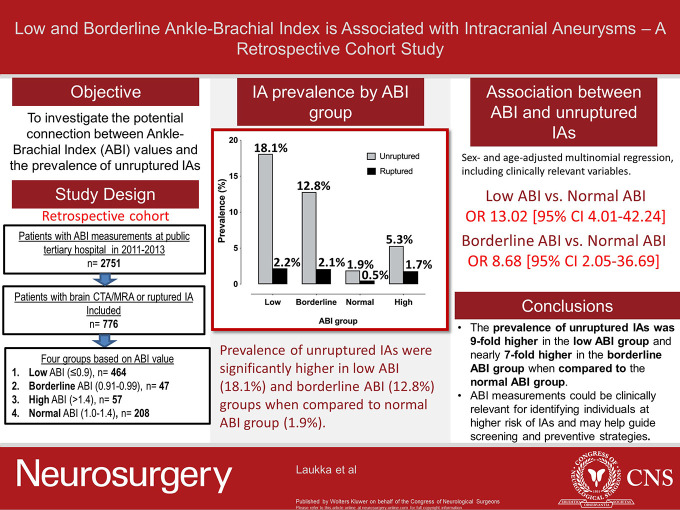

**ABBREVIATIONS:**
ABI: Ankle–brachial index
IA: intracranial aneurysm
MRA: magnetic resonance angiography.

The prevalence of unruptured intracranial aneurysms (IAs) in the general population is approximately 3%,^1^ and the annual incidence of aneurysmal subarachnoid hemorrhage is approximately 8 per 100 000.^2^ Hypertension and smoking are the most important modifiable risk factors for IAs.^3,4^ Higher atherosclerotic burden may be associated with an increased risk of IAs,^5,6^ and IAs may be linked to excess mortality because of cardiovascular diseases.^7,8^

The ankle–brachial index (ABI) is an easily available method for confirming the diagnosis of peripheral artery disease. An ABI ≤0.9 and >1.4 is considered abnormal, whereas ABIs falling within a range of 0.91–0.99 are considered borderline.^9^ A low ABI (≤0.9) and a borderline ABI are associated with an increased risk of cardiovascular diseases and stroke.^10,11^ ABI can also be used to assess cardiovascular disease risk independently of other risk factors.^12^ A low ABI may be associated with an increase in the risk of abdominal aortic aneurysms,^13,14^ whereas aortic aneurysms may be associated with cerebral aneurysms^15^ and vice versa.^16^ The ABI further serves as a measure of systemic atherosclerosis.^17^

Despite the known predictive value of ABI for cardiovascular disease and potentially for aortic aneurysms, there have been no published studies that have investigated the association between ABI and IAs as far as the authors are aware. The objective of this study is to evaluate whether there is an association between ABI and IAs.

## METHODS

The study was approved by the institutional review board. Patient consent was not required because of the retrospective nature of the study.

### Population

All patients (n = 2757) who had ABI determined in the vascular laboratory at the Department of Clinical Physiology, at public tertiary hospital, from 1 January 2011, to 31 December 2013, were reviewed retrospectively. The vascular laboratory provides noninvasive pressure measurement covering the hospital region catchment area population of 480 000 inhabitants.

Radiological examinations and electronic patient charts were reviewed until 1 January 2023.

Of the 2757 patients, 776 with available imaging studies (magnetic resonance angiography, computed tomography angiography, or digital subtraction angiography) or a history of ruptured IA were included to the further analysis.

### Baseline Measurements

The following variables were obtained from the electronic patient records: smoking history (current/former smoker, never smokers); hypertension (if the patient had diagnosed hypertension and/or antihypertensive medication); hypercholesterolemia (if the patient had diagnosed hypercholesterolemia and/or medication for hypercholesterolemia); diabetes type 1; diabetes type 2 (diagnosed diabetes type 2 and/or medication for diabetes type 2); coronary artery disease (if the patient had diagnosed coronary artery disease, previous artery bypass surgery or angioplasty, or previous myocardial infarction); malignancy (previous diagnosis of any malignancy); chronic obstructive pulmonary disease (if the patient had diagnosed chronic obstructive pulmonary disease); rheumatoid arthritis (if the patient had diagnosed rheumatoid arthritis); and varicose ulcus (if the patient had diagnosed varicose ulcus).

Qualified sonographers measured systolic blood pressure from the posterior tibial and dorsalis pedis arteries in both legs, as well as systolic brachial pressure from both arms. The higher ankle pressure value and the higher arm pressure value were used to calculate ABI. The lower value of the bilateral ABI determinations was used for the analysis.

One of the authors (consultant neurosurgeon) evaluated all cerebrovascular imaging studies for IAs. A neuroradiologist, with over 10 years of experience, evaluated every IA to confirm the diagnosis. Any disagreements in the evaluations between the neurosurgeon and the neuroradiologist were resolved through consensus.

IAs that were saccular and larger than or equal to 2 mm in size and located intracranially were considered as an IA. IA size was determined for the largest unruptured IA or for the ruptured IA.

The location of IA was recorded for the largest unruptured IA or for the ruptured IA. According to the Bouthillier classification,^18^ aneurysms located distal to the clinoid segment (C5) were defined as intradural, whereas aneurysms located in the intracavernous segment (C4) were classified as extradural. Aneurysms in and proximal to the C4 segment were not analyzed. Unruptured IAs located in the internal carotid artery were categorized as follows: ophthalmic artery, posterior communicating artery, anterior choroidal artery, and internal carotid artery bifurcation aneurysms. Middle cerebral artery aneurysms (MCA) were categorized as M1-segment, M1 bifurcation, M1/M2 bifurcation, M2, and M3–M4 segments. Anterior cerebral artery aneurysms were categorized as A1-segment, anterior communicating artery, and distal anterior cerebral artery aneurysms. Finally, posterior circulation artery aneurysms were categorized as posterior cerebral artery P1, P2, and P3, basilar tip, basilar trunk, vertebral artery, superior cerebellar artery, posterior inferior cerebellar artery, and anterior inferior cerebellar artery.

### Statistical Methods

The primary outcome measure was the prevalence of both unruptured and ruptured IAs, either together or separately.

Patients were classified into 4 groups based on their ABI values: low ABI (≤0.9), borderline ABI (0.91-0.99), normal ABI (1.0-1.4), and high ABI (>1.4). According to the presence of IA, patients were further categorized into 3 groups: those with ruptured IAs, those with unruptured IAs, and those without IAs. Patients with both ruptured IAs and unruptured IAs were classified into the ruptured IA group. Smoking history was categorized into “yes” (comprising current or former smokers) and “no” (never smokers). Missing data of smoking were not included in the multinomial regression analysis.

Associations between the different groups were calculated using the χ^2^ test for categorical data. For continuous data, Kruskal–Wallis tests were used for nonparametric data and one-way analysis of variance for normally distributed data.

Multinomial regression analyses were performed to evaluate the association of ABI groups with the IAs. The results were presented as adjusted odds ratios (ORs) with 95% CI. In the first set of calculations, adjustments were made for clinically relevant variables. In the second set of calculations, only variables that showed statistically significant values (*P <* .05) in the univariate analysis were included in the multivariate analysis. Similarly, the associations between ABI groups and covariates were calculated. Both models were adjusted for sex and age.

Continuous data are expressed as either the mean ± SD or the median IQR depending on their distribution. Missing data were excluded from the analysis.

Data were analyzed using the JMP16 statistics package (SAS Institute). A *P* value below .05 was considered statistically significant.

## RESULTS

**FIGURE 1.**
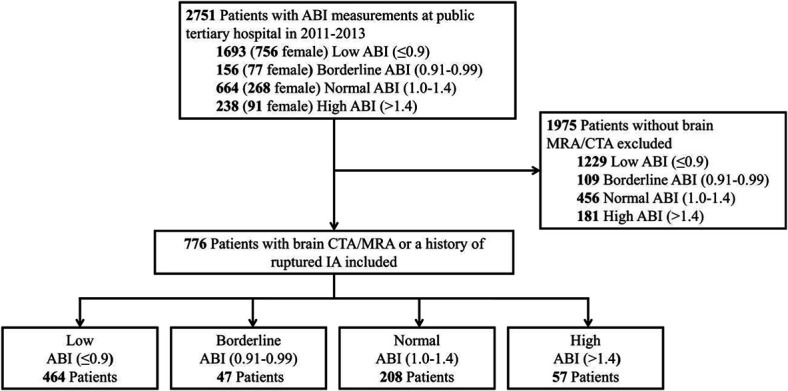
Flow diagram of the study population. ABI, ankle–brachial index; CTA, computed tomography angiography; IA, intracranial aneurysm; MRA, magnetic resonance angiography.

Figure 1 illustrates the selection of the study population.

Among the 2751 patients who underwent ABI measurements, 1693 (61.5%) exhibited a low ABI (≤0.9), 156 (5.7%) had a borderline ABI (0.91-0.99), 238 (8.7%) presented with a high ABI (>1.4), and 664 (24.1%) demonstrated a normal ABI (1.0-1.4).

Of these 2751 patients, 776 were included in the study. All of them had undergone brain magnetic resonance angiography (MRA) and/or computed tomography angiography (CTA) or had a confirmed diagnosis of ruptured IA.

The imaging rate (brain MRA/CTA) stratified by ABI group was as follows: 27.4% (464 of 1693) in the low ABI group, 30.1% (47 of 156) in the borderline ABI group, 23.9% (57 of 238) in the high ABI group, and 31.3% (208 of 664) in the normal ABI group.

A total of 5 patients underwent brain MRA/CTA screening for unruptured IAs: 1 patient in the normal ABI (1.0-1.4) group, 3 in the low ABI (≤0.9) group, and 1 in the borderline ABI (0.91-0.99) group.

Of the 776 individuals included in the study, 464 (59.8%) had a low ABI (≤0.9), 47 (6.1%) had a borderline ABI (0.9177-0.99), 57 (7.3%) had a high ABI (>1.4), and 208 (26.8%) had a normal ABI (1.0-1.4).

Regarding cerebrovascular imaging analyses, there were no discrepancies in the diagnosis of IAs between the 2 interpreting physicians.

### Associations of ABI Groups With Intracranial Aneurysms

**FIGURE 2.**
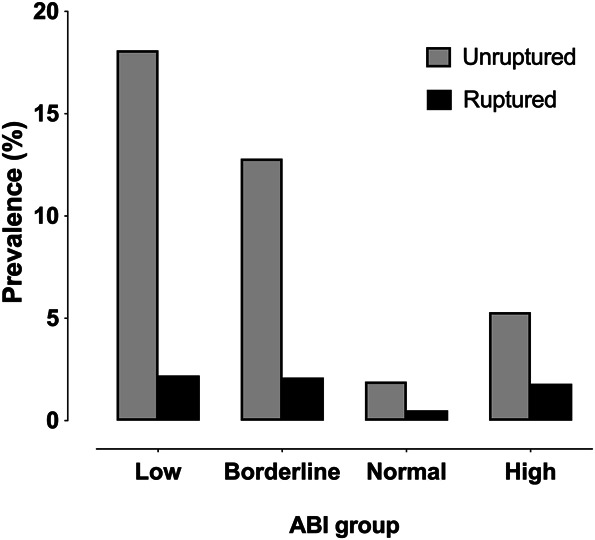
Prevalence of unruptured and ruptured intracranial aneurysms by ABI group. ABI, ankle–brachial index.

Table 1 presents the main baseline demographics of patients and IAs categorized by ABI groups. Figure 2 presents the prevalence of unruptured and ruptured IAs by ABI groups.

**TABLE 1.** Comparison of Baseline and IA Characteristics According to the ABI Group

Variable	Normal ABI (1.0-1.4) *n* = 208	Low ABI (≤0.9) *n* = 464	Borderline ABI (0.91-0.99) *n* = 47	High ABI (>1.4) *n* = 57	*P* value
Baseline characteristics					
Mean age at ABI measurement, y, SD	67.0 ± 10.8	69.8 ± 9.6	66.1 ± 13.1	68.3 ± 11.5	**.003**
Mean age at cerebrovascular imaging, y, SD	69.4 ± 11.9	72.2 ± 10.0	68.6 ± 14.2	68.0 ± 12.6	**.010**
IA screening, n (%)	3 (1.4%)	1 (0.2%)	1 (2.1%)	0 (0%)	.146
Sex (female) (%)	39.4%	38.4%	53.2%	22.8%	**.016**
Smoking history, No. of patients, total					**<.001**
Yes	55.1%	78.6%	68.9%	58.9%	
No	44.9%	21.4%	31.1%	46.1%	
Smoking history, missing data (%)	6.7%	6.3%	4.3%	8.8%	.815
Hypertension (%)	60.1%	66.0%	59.6%	75.4%	.129
Diabetes type 1 or 2 (%)	35.1%	36.9%	46.8%	63.2%	**<.001**
Diabetes type 1 (%)	7.2%	3.9%	19.2%	15.8%	**<.001**
Diabetes type 2 (%)	27.9%	33.3%	25.5%	50.9%	**.008**
Hypercholesterolemia (%)	24.0%	30.5%	27.7%	36.8%	.19
Coronary artery disease (%)	29.8%	36.0%	34.0%	42.1%	.27
Chronic heart failure (%)	13.9%	16.4%	21.3%	28.1%	.069
Atrial fibrillation	26.0%	19.4%	29.8%	36.8%	**.008**
Chronic kidney failure (%)	10.6%	11.9%	19.2%	33.3%	**<.001**
Chronic obstructive pulmonary disease (%)	6.3%	14.7%	12.8%	14.0%	**.022**
Rheumatoid disease	11.1%	7.3%	6.4%	10.5%	.371
Varicose ulcus	7.2%	5.6%	8.5%	14.0%	.112
Malignancy	17.8%	19.4%	12.8%	31.6%	.070
Intracranial aneurysm characteristics					
Prevalence of IAs, total No. of patients (%)	5 (2.4%)	94 (20.3%)	7 (14.9%)	4 (7.0%)	**<.001**
Unruptured, n (%)	4 (1.9%)	84 (18.1%)	6 (12.8%)	3 (5.3%)	**<.001**
Ruptured, n (%)	1 (0.5%)	10 (2.2%)	1 (2.1%)	1 (1.7%)	.277
Prevalence of unruptured IA by sex, No. of patients (%)					.504
Female	2 (2.4%)	34 (19.1%)	4 (16.0%)	2 (15.4%)	
Men	2 (1.6%)	50 (17.5%)	2 (9.1%)	1 (2.3%)	
Proportion of multiple IAs, No. of patients (%)					.213
Among unruptured IA	2 (50%)	9 (10.7%)	0%	0%	
Among ruptured IA	0%	5 (50%)	0%	0%	
Median size of the largest/ruptured IA, mm (IQR)	4.0 (3.0-5.0)	4.0 (3.0-5.0)	3.0 (3.0-8.0)	4.0 (3.0-7.0)	.929
Distribution of largest or ruptured IA					.817
Anterior cerebral artery	20.0%	25.8%	28.6%	50.0%	
Internal carotid artery	60.0%	31.2%	14.3%	25.0%	
Middle cerebral artery	20.0%	21.5%	28.6%	0%	
Posterior circulation	0%	8.6%	28.6%	0%	
Intracavernous internal carotid artery	0%	12.9%	0%	25.0%	

ABI, ankle–brachial index; IA, intracranial aneurysm.

Values in bold indicate statistically significant value *P <* .05. Patients with both ruptured IA and unruptured IA were categorized as having ruptured IA.

The prevalence of unruptured IAs was 18.1% in the low ABI (≤0.9) group, 12.8% in the borderline ABI (0.91-0.99) group, 5.3% in the high ABI (>1.4) group, and 1.9% in the normal ABI (1.0-1.4) group (*P <* .001). Notably, 1 patient in the low ABI (≤0.9) group was identified with an unruptured IA through screening and managed conservatively.

Within the low ABI (≤0.9) group, 2.2% had ruptured IAs, whereas in the borderline ABI (0.91-0.99) group, 2.1% had ruptured IAs. In the high ABI (>1.4) group, 1.8% had ruptured IAs, and in the normal ABI (1.0-1.4) group, 0.5% had ruptured IAs (*P =* .277).

Table 2 displays the main baseline demographics of patients categorized by IA presentation. The median ABI was 0.59 (IQR 0.45-0.75) in patients with unruptured IAs, 0.57 (IQR 0.51-0.79) in patients with ruptured IAs, and 0.80 (IQR 0.53-1.13) in patients without unruptured IAs (*P <* .001).

**TABLE 2.** Baseline Characteristics of Unruptured and Ruptured IAs and Patients Without IAs

Variable	Unruptured IA n = 97	Ruptured IA n = 13	Without IA n = 666	*P* value
Age, y, mean (SD)	69.5 (8.7)	68.9 (8.9)	68.6 (10.6)	.753
Sex (female) (%)	43.3%	46.1%	37.5%	.467
Multiple IAs	11.3%	38.5%	—	**.022**
Smoking history (%)				**.013**
Yes	82.8%	61.5%	68.2%	
No	17.2%	38.5%	31.8%	
Smoking history, missing data (%)	4.1%	0%	6.9%	.229
Hypertension (%)	68.0%	76.9%	64.0%	.477
Diabetes type 1 or 2 (%)	39.2%	15.4%	39.4%	.213
Diabetes type 1 (%)	5.2%	0%	6.9%	.508
Diabetes type 2 (%)	34.0%	15.4%	32.8%	.396
Hypercholesterolemia (%)	34.0%	30.8%	28.3%	.508
Coronary artery disease (%)	29.9%	7.7%	35.9%	.061
Chronic heart failure (%)	19.6%	0.0%	16.8%	.207
Atrial fibrillation	16.5%	7.7%	24.3%	.096
Chronic kidney failure (%)	15.5%	15.4%	13.2%	.820
Chronic obstructive pulmonary disease	18.6%	7.7%	11.4%	.118
Rheumatoid disease	6.2%	23.1%	8.6%	.121
Varicose ulcus	6.2%	23.1%	6.6%	.064
Malignancy	17.5%	7.7%	20.0%	.475
ABI, median (IQR)	0.59 (0.45-0.75)	0.57 (0.51-0.79)	0.80 (0.53-1.13)	**<.001**

ABI, ankle–brachial index; IA, intracranial aneurysm.

Values in bold indicate statistically significant value *P <* .05.

Multinomial regression analyses, adjusted for age, sex, and clinically significant variables (Table 3, Model 1), revealed that low ABI (≤0.9) (OR, 13.02; 95% CI, 4.01-42.24; *P <* .001), borderline ABI (0.91-0.99) (OR, 8.68; 95% CI, 2.05-36.69; *P =* .003), and smoking history (OR, 2.01; 95% CI, 1.07-3.77; *P =* .030) were the only variables associated with unruptured IAs. Similar results were observed in the second model (Table 3, Model 2), which were adjusted for age, sex, and statistically significant variables.

**TABLE 3.** Multinomial Regression Models for Unruptured IAs Adjusted With Sex and Age

Variable	OR	95% CI	*P* value
Model 1			
Age	1.02	0.99–1.04	.209
Sex (female vs male)	1.53	0.95–2.46	.083
Hypertension	1.23	0.75–2.02	.417
Smoking history (yes vs no)	2.01	1.07–3.77	**.030**
Coronary artery disease	0.61	0.37–1.02	.058
Chronic kidney failure	1.46	0.74–2.84	.273
Normal ABI 0.9–1.4	ref.		
Low ABI ≤0.9	13.02	4.01–42.24	**<.001**
Borderline ABI	8.68	2.05–36.69	**.003**
High ABI >1.4	3.97	0.76–20.76	.103
Model 2			
Age	1.01	0.99–1.04	.295
Sex (female vs male)	1.58	0.99–2.54	.056
Smoking history (yes vs no)	1.97	1.05–3.68	**.035**
Normal ABI 0.9–1.4	ref.		
Low ABI ≤0.9	12.69	3.91–41.12	**<.001**
Borderline ABI	8.80	2.09–36.98	**.003**
High ABI >1.4	4.27	0.83–22.00	.083

ABI, ankle–brachial index; IA, intracranial aneurysm; OR, odds ratio.

Model 1 included clinically significant variables. Model 2 included variables that were statistically significant in the univariate analysis. Values in bold indicate statistically significant value *P <* .05.

### Associations With ABI Groups

The multinomial regression analysis revealed that age (OR, 1.06; 95% CI, 1.04-1.08; *P <* .001), smoking history (OR, 4.19; 95% CI, 2.77-6.34; *P <* .001), and atrial fibrillation (OR, 0.59; 95% CI, 0.38-0.91; *P* = .018) were associated with low ABI (≤0.9). Female sex (OR, 2.18; 95% CI, 1.10-4.31; *P =* .026) and smoking history (OR, 2.35; 95% CI, 1.11-4.96; *P =* .025) were associated with borderline ABI (0.91-0.99). Diabetes type 1 or 2 (OR, 2.73; 95% CI, 1.36-5.49; *P =* .005) and chronic kidney failure (OR, 3.96; 95% CI, 1.79-8.76; *P <* .001) were associated with high ABI (>1.4), while females had a reduced risk (OR, 0.39; 95% CI, 0.17-0.88; *P =* .024) of high ABI (>1.4). Refer to **Supplemental Digital Content 1** (http://links.lww.com/NEU/E90) for further details.

## DISCUSSION

We found that both low ABI (≤0.9) and borderline ABI (0.91-0.99) were significantly associated with an increased risk of IAs when compared with patients with a normal ABI (1.0-1.4). The prevalence of unruptured IAs in the low ABI group (≤0.9) was 18.1%, whereas the prevalence of ruptured IAs was 2.2%. Similarly, in the borderline ABI group, the prevalence of unruptured IAs was 12.8%, and ruptured IAs were found in 2.1% of the cases. By contrast, individuals with a normal ABI had a much lower prevalence of unruptured IAs (1.9%) and ruptured IAs (0.5%). This pattern of findings suggests a vastly increased risk of IAs in patients with reduced ABI.

Our findings demonstrate a 9-fold higher prevalence of unruptured IAs in the low ABI group and approximately 7-fold higher prevalence in the borderline ABI group compared with those with a normal ABI range of 1.0–1.4. Notably, the prevalence of unruptured IAs in the normal ABI group was similar to that reported in the general population.^1^

The prevalence of unruptured IAs in the low ABI group and borderline ABI group was similar to that observed in specific populations known to have a high risk of IAs, such as patients with polycystic kidney disease or those who have at least 2 first-degree relatives with IAs.^19^ Consequently, screening for IAs is recommended in these high-risk populations.^19^ In addition, female smokers have been reported to exhibit a prevalence of IAs ranging from 12%^20^ to 19%.^21^

Low ABI (≤0.9)^22,23^ and high ABI (>1.4)^24^ serve as markers of vascular disease and can predict cardiovascular mortality beyond known cardiovascular risk factors. Low ABI correlates well with other indicators of systemic atherosclerosis, such as coronary artery calcification^25,26^ and abdominal aortic calcification,^26^ and it does not require imaging investigation/exploration, thus making it a readily available indicator. Moreover, IAs may be associated with an increased burden of atherosclerosis.^5,6^ However, the link between cardiovascular diseases and IAs has hitherto received relatively little attention.^7,8^

Hypertension and smoking are shared risk factors for IAs^3,4^ and low/borderline ABI,^27^ which may partly explain our findings. In our study, smoking history, low ABI, and borderline ABI each emerged as independent risk factors for IAs. However, in the multinomial regression, the relationship between smoking and IAs appeared to be comparatively weaker than that of low ABI and borderline ABI. This suggests that low ABI and borderline ABI may be associated with IAs through different underlying mechanisms. Low ABI is indicative of a combination of several different risk factors,^17^ including genetics,^17^ and serves as an objective marker of vascular disease irrespective of other risk factors.^22,23^ Low ABI may be associated with systemic inflammation and endothelial dysfunction,^28^ which could increase the risk of IAs.^29^

In our investigation, we found no statistically significant difference in the prevalence of unruptured intracranial IAs between females and males in the different ABI groups. However, there was an observed trend toward a heightened risk of unruptured IAs in females, and the inclusion of a larger sample size might have potentially revealed a significant difference. Consistent with earlier research, it has been reported that in the general population, females exhibit a 1.5–2 times higher prevalence of unruptured IAs when compared with males.^1,30,31^ This suggests the possibility that distinct risk factors for IAs may exist between females and males,^32^ factors that may not necessarily be reflected in ABI.

A prospective study will be required to making recommendations about IA screening based on ABI. Nonetheless, our study data show that the prevalence of IAs in low ABI and in borderline ABI groups is exceptionally high. Beyond the established criteria for IA screening, the ABI emerges as a potential, straightforward screening tool for identifying patients at elevated risk of IAs. In our study, only 5 patients underwent imaging because of IA screening indications, and thus, our findings do not provide a conclusive answer regarding the utility of ABI in individuals with recognized IA screening indications. Consequently, those presenting with such indications should undergo screening according to prevailing standards, irrespective of a normal ABI.

Our population was too small to draw conclusions about whether there are differences between ABI groups regarding ruptured IAs. Yet, despite there being no statistically significant difference, only 0.5% of patients had ruptured IAs in the normal ABI group in contrast to the low ABI and borderline ABI groups, both of which had around 2% ruptured IAs. It is intuitive to think that a population with a high prevalence of unruptured IAs may be at higher risk for ruptured IAs.

### Limitations

We acknowledge the limitations of our study. It is important to note that this retrospective study may introduce potential selection bias. Of the original study population of 2757 patients, only 32% underwent cerebrovascular imaging or had ruptured IAs, and they were included in our analysis. The reasons for undergoing imaging could vary, which might lead to a possible selection bias. However, it is also worth mentioning that we had a comprehensive population, particularly for the low ABI and normal ABI groups. Despite this, the prevalence of IAs was exceptionally high in the low and borderline ABI groups, which might suggest that selection bias alone cannot explain our findings.

We were able to collect smoking history data in 94% of patients and missing data of smoking history had similar distribution among different ABI groups. Because of the retrospective nature of this study, more comprehensive data of smoking status, for example, pack years, can be challenging to get from the electronic health records.^33^

## CONCLUSION

The prevalence of unruptured IAs was nearly 9-fold higher in the low ABI group and nearly 7-fold higher in the borderline ABI group compared with the normal ABI group. Notably, the prevalence of unruptured IAs in the normal ABI group was similar to that reported in the general population. ABI measurements could be clinically relevant for identifying individuals at higher risk of IAs and may help guide screening and preventive strategies in addition to established criteria for IA screening.

## Supplementary Material

## SUPPLEMENTAL DIGITAL CONTENT

**Supplemental Digital Content 1. Table 1.** Risk factors for different Ankle-Brachial Index (ABI) groups. Multinomial regression analysis was adjusted with sex and age, and included variables that were statistically significant in univariate analysis. Values are ORs (95% CI). Bold indicate statistically significant value *P <* .05.

## COMMENTS

Laukka et al present the first known evidence of a statistical correlation between ABI and the presence of intracranial aneurysms. As a surrogate for severity of systemic vascular disease, this association is perhaps unsurprising, but the strength of the association is notable. In a large cohort of patients, low ABI was associated with 9-fold increased risk of aneurysm incidence, and borderline ABI was associated with a 7-fold increase. With focus on the low-ABI cohort, the authors have identified a patient population with 18% incidence of unruptured aneurysms. For reference, autosomal dominant polycystic kidney disease is associated with 12%–20% risk of intracranial aneurysms and, for this reason, is widely considered an indication for noninvasive screening. Although this study alone may not warrant universal expansion of screening practices, it adds a powerful tool to the arsenal of cerebrovascular specialists treating an otherwise rare disorder.

**Peter Theiss and Ali Alaraj**

*Chicago, Illinois, USA*

## Notes

It is well known that some underlying diseases, certain genetical dispositions, and modifiable risk factors such as hypertension and smoking are linked to a high prevalence of intracranial aneurysms (IAs). In contrast, for the first time, the present paper documents an association between the calculated ankle–brachial blood pressure index (ABI) and the prevalence of IAs. In their study, the authors found that 18.1% of their 464 patients with low ABI and 12.8% of 47 patients with borderline ABI harbored unruptured IAs compared with 1.9% of the 208 patients with normal ABI, ie, a remarkable 9-fold and almost a 7-fold increase in unruptured IAs among those with low ABI and borderline ABI, respectively. Differences in smoking habits between the patient groups may explain only a fraction of these large differences in prevalence. Although differences in the prevalence of ruptured IAs between patient groups could have emerged given a larger patient cohort, this remains to be delineated.

The ABI is used not only for diagnosing peripheral arterial disease but also serves as a measure of the atherosclerotic state of an individual. With low ABI being linked to a high prevalence of abdominal aortic aneurysms as well as cardiovascular events such as myocardial infarction and stroke, the present finding of an association between low ABI and high prevalence of unruptured IAs comes as no big surprise. It may instead be looked upon as one more “piece of the puzzle” in the linkages between the various diseases of the arterial tree.

If the findings of the present study can be confirmed in a prospective study, a low ABI could be added to the established criteria for intracranial aneurysm screening. I congratulate the authors for their novel finding.

**Wilhelm Sorteberg**

*Oslo, Norway*
