# Supplementary material for: Low and Borderline Ankle–Brachial Index Is Associated With Intracranial Aneurysms: A Retrospective Cohort Study
Source: Neurosurgery. 2024 Jan 25;94(6):1282–90. doi: 10.1227/neu.0000000000002837 (PMC11073771; doi:10.1227/neu.0000000000002837)
Supplement: Supplementary file 1 [file neu-94-1282-s001.docx]

**Supplemental Table 1.** Risk factors for different ankle-brachial index (ABI) groups. Multinomial regression analysis was adjusted with sex- and age, and included variables that were statistically significant in univariate analysis. Values are odds ratios (95% CI). Bold indicate statistically significant value *p<*0.05.

| **Variable** | **Low ABI** | **Borderline ABI** | **High ABI** |
| --- | --- | --- | --- |
| Age | **1.06 (1.04-1.08)** | 1.01 (0.98-1.04) | 1.02 (0.99-1.06) |
| Sex (female vs. male) | 1.23 (0.84-1.80) | **2.18 (1.10-4.31)** | **0.39 (0.17-0.88)** |
| Diabetes type 1 or 2 | 1.15 (0.78-1.69) | 1.54 (0.77-3.09) | **2.73 (1.36-5.49)** |
| Atrial fibrillation | **0.59 (0.38-0.91)** | 1.19 (0.54-2.63) | 1.66 (0.82-3.35) |
| Chronic kidney failure | 1.34 (0.73-2.47) | 2.06 (0.82-5.21) | **3.96 (1.79-8.76)** |
| Smoking history (yes vs. no) | **4.19 (2.77-6.34)** | **2.35 (1.11-4.96)** | 1.00 (0.51-1.97) |
